# Opioid-Prescribing Trends in Dermatology From 2014 to 2020 in the United States

**DOI:** 10.7759/cureus.74425

**Published:** 2024-11-25

**Authors:** Anastasia Smirnoff, Santiago Rengifo, Henley Spracklen, Erum N Ilyas

**Affiliations:** 1 Dermatology, Drexel University College of Medicine, Philadelphia, USA; 2 Foundation for Opioid Research and Education, Rothman Orthopaedic Institute, Philadelphia, USA

**Keywords:** clinical dermatology, opioids, prescribing patterns, prescription opioids, public data

## Abstract

Introduction

The opioid epidemic is a critical public health crisis, with opioid overdose deaths being a leading cause of injury-related deaths in the United States. Dermatology, though a small contributor to overall opioid prescriptions, still accounts for over 700,000 opioid pills annually. Reducing opioid prescribing in this specialty has been challenging due to limited comprehensive research. This study aimed to investigate opioid-prescribing trends in dermatology across all nine US regions over seven years.

Methods

Data on opioid prescriptions by dermatologists from 2014 to 2020 were collected retrospectively from the Medicare Part D Prescribers by Provider database, available through the United States Centers for Medicare and Medicaid Services (CMS). The data were analyzed nationally and by geographic division, using US census population estimates for the respective states to calculate rates per population. Over the years studied, opioid prescription claims, the number of dermatologists, and the proportion of dermatologists prescribing opioids gradually decreased both by average and by population.

Results

Over the years evaluated, there were a total of 618,714 claims for short-acting opioids throughout the United States. Prescription claims, the number of dermatologists, and the proportion of dermatologists prescribing opioids all saw a gradual decrease in numbers by average and by population. Claims per year decreased from 2,023 in 2014 to 1,124 in 2020. Dermatologists per 10,000 people decreased from 0.35 in 2014 and 2015 to 0.32 in 2020. The percentage of dermatologists prescribing opioids decreased from 16.5% (0.06 per 10,000 people) in 2014 to 9.14% (0.03 per 10,000 people) in 2020. Over the seven-year period, the geographic state divisions that make up the south region had the most claims by population with 3330 claims in division 5 (3.9 per 10,000 people), 2531 claims in division 6 (5.2 per 10,000 people), and 3287 claims in division 7 (2.97 claims per 10,000 people). Division 1 had the least amount, with 0.6 claims per 10,000 people.

Conclusion

The findings show a gradual decline in opioid prescriptions by dermatologists, consistent with the national trend. Moreover, there are significant regional variations in opioid prescribing, with the southern states having the highest prescribing rates. The study highlights the need for targeted education policies to address regional variations and promote standardized opioid protocols in dermatology practice.

## Introduction

The opioid epidemic has become one of the most extensive public health crises, with opioid overdose deaths now ranked as a leading cause of injury-related death in the United States [1]. The first wave of the opioid epidemic was driven by aggressive opioid overprescribing and saw prescription opioid sales increase by over 400% between 1999 and 2011, with a subsequent increase in opioid-related deaths. Following the official declaration of the opioid epidemic as a public health emergency in 2016, massive efforts have been enacted to decrease the prescription of opioids by healthcare providers in all medical specialties.

Dermatology is the medical specialty concerned with the medical and surgical treatment of skin diseases [2]. Although dermatology represents a relatively small percentage of the total opioid prescribers, the specialty still contributes to over 700,000 opioid pills that may remain unused every year [2]. Most opioids prescribed by dermatologists are following Mohs micrographic surgeries and conventional excisions of pre-cancerous and cancerous lesions [2]. Usually, acute postoperative dermatologic treatment can be managed with a combination of NSAIDs and acetaminophen. However, finding the appropriate individualized pain management protocol can still be challenging for providers [3]. While only around 10% of patients require postoperative opioid analgesics following dermatologic procedures, approximately 7000 patients annually will continue to use opioids for at least one year following dermatological surgeries [4].

The short-term utilization of opioids following any surgical procedure can put patients at risk of long-term use [5]. Thus, postoperative opioid use following dermatologic surgery can expose opioid-naive patients to these medications, increasing the risk of addiction even when taken short term [6]. Despite updates in opioid-prescribing guidelines among many medical and surgical specialties, opioid-related research in the field of dermatology is limited [2,4].

Few reports exist regarding opioid-prescribing trends in dermatology within the United States. The information available has reported wide variations in the number of opioid prescriptions from state to state, with dermatologists in the southern states prescribing more opioids than dermatologists in the rest of the country [6]. However, there are currently no reports that examine trends in opioid prescribers and prescribing rates throughout the entire United States over an extended time period and analyze how these rates have been changing over time. This study investigated opioid-prescribing trends within dermatology in all nine US regions over a seven-year time period.

## Materials and methods

The study utilized data from the Medicare Part D Prescribers by Provider database, which is accessible through the United States Centers for Medicare and Medicaid Services (CMS) [7]. The timeframe for the data collection spanned from January 1, 2014, to December 31, 2020. The analysis encompassed dermatologists across all 50 states and the District of Columbia, providing a comprehensive overview of opioid prescription practices.

To facilitate regional comparisons, the states were categorized into nine geographic divisions according to the US Census Bureau's classification: New England, Middle Atlantic, East North Central, West North Central, South Atlantic, East South Central, West South Central, Mountain, and Pacific [8]. Population estimates from the US census for each state in the chosen year were employed to calculate rates per population [9].

The analysis included various parameters, such as the percentage of dermatologists prescribing opioids, the total number of claims in each state, and the overall count of prescribers. The data were examined both at a national level and within the specified geographic divisions. The findings were descriptively presented, reporting percentages or means and providing valuable insights into the opioid prescription landscape among dermatologists across the United States.

## Results

From 2014 to 2020, there were a total of 618,714 claims for short-acting opioids throughout the United States. Nationally, the average number of claims throughout all the years analyzed was 1733, with an average of 2.39 claims per 10,000 people (Table 1).

**Table 1:** Average values for the parameters measured over the entire seven-year period Note: N=357 is from seven years of data for 50 states and the District of Columbia SAO: short-acting opioids

	Total (SD) (n=357)
Total SAO claims	1733 (2480)
Total SAO claims per 10,000 people	2.39 (2.34)
Dermatologist	233 (297)
Dermatologist per state per 10,000 people	0.34 (0.13)
Prescribers	27.8 (36.1)
Prescribers per 10,000 people	0.04 (0.02)
Percent of dermatologists prescribing opioids	12.8 (8.64)
Percent of dermatologists prescribing opioids per 10,000 people	0.04 (0.05)

By year, 2014 to 2020

**Figure 1:**
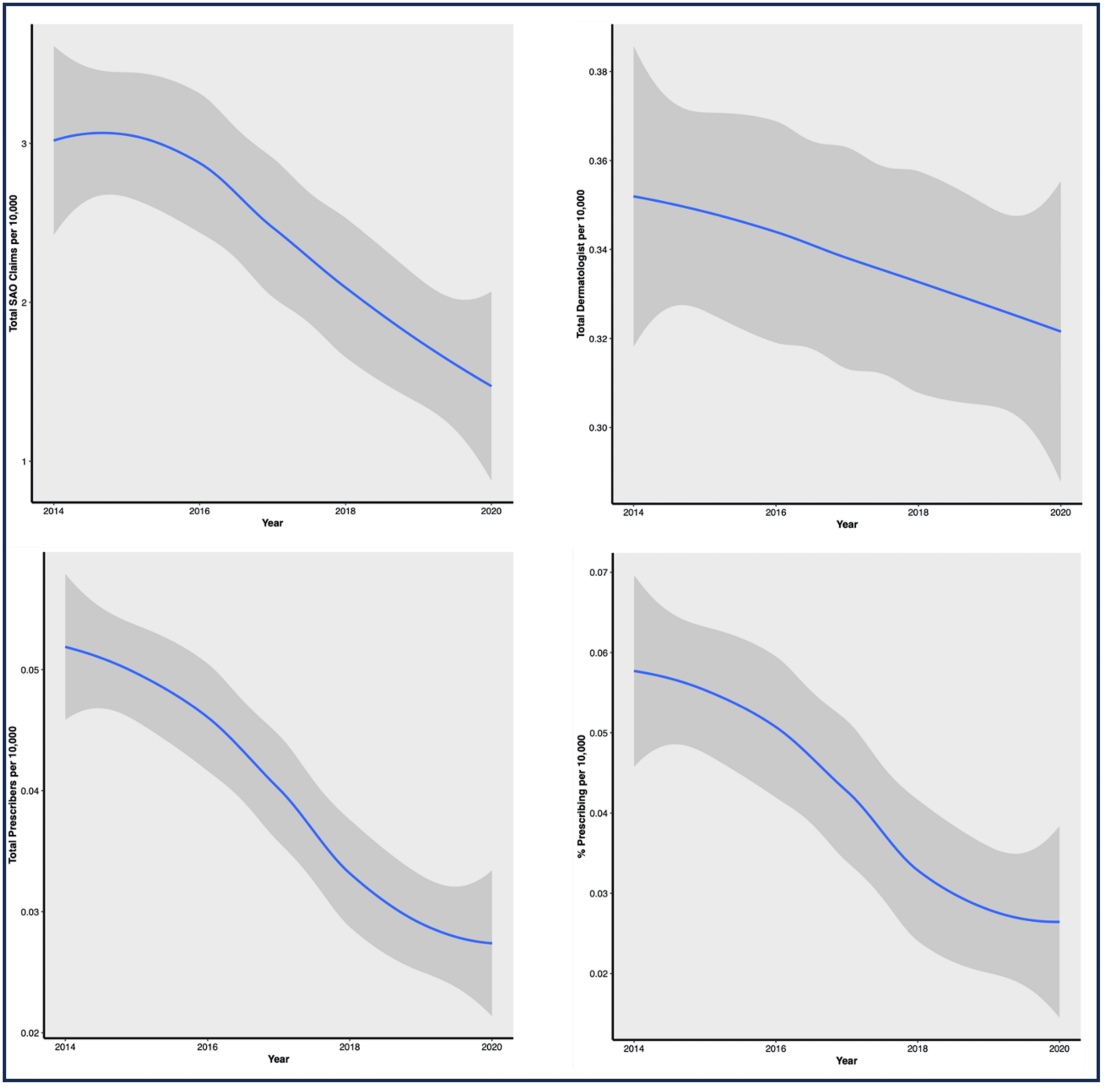
Change in parameters from 2014 to 2020 SAO: short-acting opioids

Nationally, the average number of claims for each year was 2023 in 2014, 2132 in 2015, 2013 in 2016, 1885 in 2017, 1545 in 2018, 1410 in 2019, and 1124 in 2020 (Table 2). When examining the claims by population, the average number of claims per 10,000 people was 2.99 in 2014, 3.14 in 2015, 2.82 in 2016, 2.52 in 2017, 2.03 in 2018, 1.79 in 2019, and 1.46 in 2020 (Table 2, Figure 1). Per state, the average number of dermatologists gradually decreased from an average of 238 in 2014 and 2015 to 227 in 2019 and 2020. Similarly, the average number of dermatologists per 10,000 people decreased from 0.35 in 2014 and 2015 to 0.32 in 2020 (Table 2, Figure 1). When evaluating dermatologists who prescribed opioids per state, the average number decreased from 35.2 (0.05 per 10,000 people) in 2014 to 20.6 (0.03 per 10,000 people) in 2020. The percentage of dermatologists prescribing opioids decreased from 16.5% (0.06 per 10,000 people) in 2014 to 9.14% (0.03 per 10,000 people) in 2020 (Table 2, Figure 1).

**Table 2:** Average state values for the parameters for each year SAO: short-acting opioids

	2014 (n=51)	2015 (n=51)	2016 (n=51)	2017 (n=51)	2018 (n=51)	2019 (n=51)	2020 (n=51)
SAO claims	2023 (2610)	2132 (2761)	2013 (2825)	1885 (2721)	1545 (2341)	1410 (2137)	1124 (1745)
SAO claims per 10,000 people	2.99 (2.69)	3.14 (2.79)	2.82 (2.51)	2.52 (2.33)	2.03 (2.07)	1.79 (1.81)	1.46 (1.42)
Dermatologist	238 (310)	238 (307)	235 (302)	233 (300)	230 (296)	227 (293)	227 (291)
Dermatologist per 10,000 people	0.35 (0.13)	0.35 (0.13)	0.34 (0.13)	0.34 (0.13)	0.33 (0.13)	0.33 (0.13)	0.32 (0.13)
Prescribers	35.2 (43.5)	33.2 (41.0)	31.5 (38.8)	28.5 (36.6)	23.5 (31.9)	21.9 (29.3)	20.6 (27.8)
Prescribers per 10,000 people	0.05 (0.03)	0.05 (0.03)	0.05 (0.02)	0.04 (0.02)	0.03 (0.02)	0.03 (0.02)	0.03 (0.02)
Percent of dermatologists prescribing opioids	16.5 (9.57)	15.3 (9.27)	15.1 (9.41)	13.1 (8.44)	10.6 (7.32)	9.89 (6.81)	9.14 (6.45)
Percent of dermatologists prescribing opioids per 10,000 people	0.06 (0.06)	0.05 (0.05)	0.05 (0.06)	0.04 (0.04)	0.03 (0.04)	0.03 (0.03)	0.03 (0.03)

By division, 2014 to 2020

The nine state divisions of the United States were evaluated for these parameters over the seven-year period. Division 6 had the most claims by population, with 5.2 claims per 10,000 people. Division 1 had the least amount, with 0.6 claims per 10,000 people. The divisions making up the south region had the highest number of claims, with 3330 claims in division 5 (3.9 per 10,000 people), 2531 claims in division 6 (5.2 per 10,000 people), and 3287 claims in division 7 (2.97 claims per 10,000 people) (Table 3). The northeast region had the most dermatologists by population, with 0.5 dermatologists per 10,000 people in division 1 and 0.44 dermatologists per 10,000 people in division 2. Divisions 6 and 7 had the fewest amount of dermatologists by population, with 0.25 dermatologists per 10,000 people. Division 2 had the lowest percentage of dermatologists prescribing opioids, with 0.005 per 10,000 people, followed by division 1 with 0.03 per 10,000 people (Table 3).

**Table 3:** Average values of parameters by US region and divisions SAO: short-acting opioids

Region	Division #	SAO claims	SAO claims per 10,000 people	Dermatologist	Dermatologist per 10,000 people	Percent of dermatologists prescribing opioids	Percent of dermatologists prescribing opioids per 10,000 people
Northeast	Division 1 (n=6)	250.3 (453.1)	0.6 (0.6)	131.5 (147.6)	0.5 (0.1)	4.4 (2.6)	0.03 (0.03)
	Division 2 (n=3)	1713.3 (482.3)	1.5 (0.75)	632.2 (274.9)	0.44 (0.05)	5.8 (2.13)	0.005 (0.003)
Midwest	Division 3 (n=5)	1480 (637.1)	1.6 (0.78)	307.6 (106.6)	0.32 (0.05)	9.89 (3.82)	0.12 (0.01)
	Division 4 (n=7)	468.3 (499)	1.4 (0.8)	98.5 (81.8)	0.3 (0.08)	12.2 (4.7)	0.07 (0.05)
South	Division 5 (n=9)	3330 (4207)	3.9 (3.5)	268 (271)	0.38 (0.17)	12.7 (7.6)	0.03 (0.03)
	Division 6 (n=4)	2531 (1425)	5.2 (2.6)	124 (47.4)	0.25 (0.04)	23.4 (7.6)	0.05 (0.02)
	Division 7 (n=4)	3287 (3219)	2.97 (0.93)	292 (319)	0.25 (0.1)	20.2 (7.06)	0.03 (0.02)
West	Division 8 (n=8)	844 (1095)	2.0 (1.9)	96.3 (82.3)	0.28 (0.09)	15.4 (11.2)	0.07 (0.07)
	Division 9 (n=5)	2213 (2303)	2.4 (1.8)	420 (619)	0.3 (0.1)	12.6 (7.5)	0.04 (0.04)

## Discussion

This study used Medicare Part D data to analyze opioid-prescribing trends in dermatology throughout the United States from 2014 to 2020. Our findings demonstrate a gradual decrease in dermatologic-related opioid prescriptions based on prescription claims, as well as a decrease in providers prescribing opioids. These results are consistent with the national trend of declining opioid prescriptions previously seen in dermatology [2] and among all medical specialties [10]. This is likely the result of multiple effective measures enacted aimed at curbing opioid prescription, such as the implementation of prescription drug monitoring programs (PDMP), provider education on opioid prescribing, and multimodal analgesia strategies. These measures have led to a 19% decrease in annual US opioid-prescribing rates from 2006 to 2017 and a 7% decrease in prescription opioid-related US death rates from 2018 to 2019 [10].

Dermatologists primarily prescribe opioids for postoperative pain after cutaneous surgery, such as Mohs micrographic surgery. Opioids may also be prescribed for pain management in all types of psoriasis with and without psoriatic arthritis as well as herpes zoster [11,12]. Taylor et al. analyzed opioid-prescribing patterns during outpatient visits for individuals with psoriasis and psoriatic arthritis in the United States from 2006 to 2016. They found that while dermatologists accounted for over half of the total visits, they were significantly less likely to prescribe opioids compared to physicians from other medical specialties [11].

Studies on pain levels and opioid use after Mohs micrographic surgery indicate that pain is highest on the day of surgery [13-15]. However, on their most painful day, patients typically report low pain scores of around 2 out of 10 on the VAS scale [14,16,17]. Consequently, the literature suggests that patients undergoing cutaneous surgery do not necessarily need opioid analgesia, as non-opioid interventions provide sufficient or even better pain control [18,19,13-15]. Additionally, in 2019, the American Academy of Dermatology Association issued recommendations for the most common dermatologic procedures stating that postprocedural pain management should be considered for only 20 procedures, most of which should receive less than 10 pills [20]. The list of procedures includes rotation, transposition, advancement flap of the scalp, ear, nose, cheek, lip, and perineum, interpolation flap of the ear, wedge repair of the ear and lip, cartilage alar-batten graft of the nose, Mustardé flap of the cheek, and nail avulsion [4]. The findings are consistent with our study, which observed a continuous decline in opioid prescriptions during the study period.

Opioid-prescribing trends are affected by the location of both prescribers and patients [21-26]. In the present study, while there was a substantial decrease in opioid prescribing among dermatologists during the years studied, there was considerable variability in prescribing patterns among dermatologists across different regions in the United States. Specifically, the southern region (divisions 5, 6, and 7) had the highest number of opioid claims and prescribing dermatologists, despite having the lowest number of dermatologists per population. On the other hand, the northeastern region (divisions 1 and 2) had the highest number of dermatologists per population but the fewest opioid claims and prescribing dermatologists. These findings align with similar studies on medical specialties in the United States, indicating that the northeastern region generally has the lowest opioid-prescribing rate per population, while the southern region tends to have the highest [20,22]. This geographical variation may be due to differences in patient and provider attitudes and beliefs and greater regulatory pressures, including licensing boards and other local regulators, in the northeast states to decrease opioid prescribing [20,21,23]. However, several studies have found regional variations in opioid-prescribing patterns that differ from these findings. For instance, a study on otolaryngologists in 2015 revealed that the Midwest had the highest number of opioid prescriptions, while another study focusing on plastic surgery between 2016 and 2018 found that the south and northeast regions had the lowest prescription rates [25]. Nevertheless, regional trends are difficult to separate from other influences likely also contributing to variations in opioid prescribing, such as patient racial-/ethnic-, socioeconomic-, and insurance-related factors, and physician-specific factors such as board certification, experience, and gender.

Overall, these data indicate that changes in attitudes toward opioid prescribing have influenced the field of dermatology, leading to a gradual decrease in opioid prescriptions by dermatologists over time. The findings highlight the need for targeted education policies rather than broad regulatory guidelines to address regional variations in prescribing. By focusing on specific areas, policymakers can establish standardized opioid protocols, effectively combating the risks of diversion and misuse. Vital contributions can be made by organizations like the American Academy of Dermatology through their role in educating providers on effective strategies to confront this issue.

This study's interpretation must consider its limitations. It focuses on opioid claims, so it doesn't cover unfilled prescriptions, unused opioids, or those acquired without a prescription. Additionally, the data for this study was obtained from the CMS database, which means it only covers patients included in Medicare Part D. Therefore, the findings cannot be generalized to the entire US population based on age and those who are uninsured or on Medicaid. Since patients over 60 years old comprise the majority of those getting Mohs micrographic surgery, which accounts for most opioids prescribed by dermatologists, our opioid-prescribing trends can still offer valuable insights into a significant portion of opioid prescriptions for dermatologic procedures. This study does not consider patient or prescriber demographic information, which could affect individual opioid-prescribing patterns. Additionally, it does not analyze the prescription reports to track the quantities of different short-acting opioids due to the absence of this data in the CMS reports. Future research could investigate combining CMS data with other prescription databases, such as the PDMP, to monitor the prescribing of specific opioid medications.

## Conclusions

Opioid prescriptions from dermatologists decreased steadily between 2014 and 2020, aligning with the nationwide trend in opioid prescribing across medical specialties. In terms of location, southern states exhibit the highest rates of opioid prescribing by dermatologists. Further research is needed to better elucidate the need for targeted education policies to address regional variations and promote standardized opioid protocols in dermatology practice.
